# Novel agents for chronic lymphocytic leukemia

**DOI:** 10.1186/1756-8722-6-36

**Published:** 2013-05-16

**Authors:** Mei Wu, Akintunde Akinleye, Xiongpeng Zhu

**Affiliations:** 1 Department of Hematology, First Hospital of Quanzhou affiliated to Fujian Medical University, Quanzhou, 362000, China; 2 Division of Hematology/Oncology, New York Medical College and Westchester Medical Center, Valhalla, NY, 10595, USA

## Abstract

Chronic lymphocytic leukemia (CLL) is a heterogeneous group of B-cell neoplasm. CLL is typically sensitive to a variety of cytotoxic agents, but relapse frequently occurs with conventional approaches. The treatment of CLL is evolving rapidly with the introduction of novel drugs, such as bendamustine, ofatumumab, lenalidomide, ibrutinib, idelalisib, veltuzumab, XmAb5574, navitoclax, dasatinib, alvespimycin, and TRU-016. This review summarizes the most current clinical experiences with these agents in the treatment of CLL.

## Introduction

Chronic lymphocytic leukemia (CLL) is a post-germinal center neoplasm characterized by clonal proliferation and accumulation of mature-appearing lymphocytes in the blood, bone marrow, lymph nodes and spleen. The CLL cells are typically B-cell in origin, and T-cell variant occurs rarely. CLL is the most common leukemia in Western countries, and it accounts for approximately one-third of all leukemias in the United States [1]. The median age at diagnosis of CLL is 70 years, and the disorder demonstrates a slight male predilection [2]. Most patients are asymptomatic and the diagnosis of CLL is frequently made on routine blood count. The leukemic cells usually coexpress CD5 and CD23, and the diagnosis can be established by the demonstration of ≥5 × 10^9^/L monoclonal cells with this phenotype in the peripheral blood even in the absence of lymphadenopahy, organomegaly, or other clinical features [3]. CLL is a heterogeneous disease as reflected by its highly variable natural history, ranging from indolent to aggressive clinical course. Some patients die within two to three years from the time of initial diagnosis, whereas others with the condition live much longer for about 10 to 20 years [4]. To provide useful prognostic information, CLL patients are stratified into prognostic groups by the Rai and Binet clinical staging systems, which appear to correlate with degree of gross tumor burden [4,5]. However, these staging systems lack accuracy to predict disease progression particularly in early stage or low risk disease. As such, adverse cytogenetic abnormalities and molecular markers are being utilized to better identify patients with more rapidly progressive disease [6,7].

CLL is typically sensitive to a variety of cytotoxic drugs, but the disease is considered incurable. Treatment is generally recommended to control symptoms and reduce bulk of disease but without substantially improving survival. Emerging understanding of the molecular pathophysiology of CLL has facilitated the development of new drugs with a view to improving clinical outcomes for this malignancy [8-10].

## Conventional approaches

In newly diagnosed patients, primary therapy with fludarabine-based therapy demonstrated high response rates. Specifically, combination regimens with fludarabine, cyclophosphamide, and rituximab (FCR), fludarabine plus rituximab (FR), are frequently used initial treatment.

In younger (<65 years) patients, FCR has been shown be superior to either fludarabine or fludarabine combined with cyclophosphamide (FC) regimens [11,12]. However, FCR is reported to be associated with higher incidence of opportunistic infections, prolonged myelosuppression and possibly increased rate of secondary malignancies. The updated results from the German CLL Study Group (GCLLSG) phase III trial of previously untreated patients with CLL showed higher response rates after primary treatment with FCR versus FC. After a median follow-up of 5.9 years, PFS in patients treated with FCR was 38% versus 27.4% in patients treated with FC regimen. The OS was also found to be superior in the FCR arm (HR 0.7; p=0.001). Development of secondary malignancies was seen in similar proportion of patients in both groups (9.9% versus 12.1%, p = 0.4). Though grade 3 or 4 neutropenia were significantly higher in the FCR group, it did not translate into an increased rate of MDS/AML in this group [13]. A retrospective analysis of 235 newly diagnosed patients with CLL treated with frontline FCR-based therapy was performed at the University of Texas, MD Anderson Cancer. Of 145 patients without prior history of cancer, 39 patients (27%) developed second cancer including non-melanoma skin, melanoma, head and neck, prostate, breast, lung, and therapy-related MDS-AML. More deaths were observed in those with second cancer as compared with patients without second cancer (49% versus 10%, p <0.05). Based on these results, it appears that second cancers in CLL patients treated with FCR are associated with an inferior survival [14]. Petra et al. reported the results of a study involving 252 patients with newly diagnosed CLL treated with either FCR or FC [15]. The frequency of prolonged cytopenia was not signicantly different between patients treated with FCR compared with those treated with FC. However, patients with prolonged cytopenia with either regimens demonstrated a higher risk to develop MDS/AML, and poor clinical outcome.

The optimal therapy for elderly patients (≥65 years) with CLL is not currently known, and this provides the rationale for multiple ongoing cooperative studies. The phase II LLC 2007 SA trial was designed to examine the tolerability and efficacy of induction therapy with abbreviated FCR followed by either observation or maintenance rituximab in CLL patients > 65 years [16]. Early results showed that the ORR after primary therapy with four cycles of oral FC combined with 6 doses of rituximab was 96.3%. High rate of grade 3 or 4 neutropenia was observed but rarely translated into significant infections. Similarly, analysis of Australian CLL5 study also demonstrated oral FC combined with rituximab to be a safe and well tolerated regimen in CLL patients ≥65 years [17].

## Role of aspirin in CLL

Addition of aspirin to salvage FCR regimen may be of value in CLL patients with relapsed/refractory disease. Aspirin is a non-steroid anti-inflammatory drug (NSAID) that not only directly targets the cyclooxygenase enzyme, but also activate the apoptotic pathway in leukemic CLL cells by promoting DNA fragmentation, caspase activation, and proteolytic cleavage of PARP. Data from a single institution retrospective study demonstrated a significantly higher PFS and OS in patients treated concurrent aspirin plus FCR as salvage therapy compared with FCR alone [18]. However, prospective randomized data are required to establish the benefit of aspirin in the treatment of CLL.

## Treatment of CLL with high risk features

Although CLL may be morphologically similar, several subtypes are being identified at the genetic and molecular level. Deletion of 17p, overexpression of ZAP-70 and CD38, *TP53* mutation, and unmutated *IGHV* are considered high-risk features in CLL, and associated with adverse prognosis. Similarly, refractoriness to fludarabine-based therapy is associated with worse outcome.

Alemtuzumab is a humanized immunoglobulin G_1_ anti-CD52 monoclonal antibody approved by the US Food and Drug Administration (FDA) as second-line therapy for CLL patients who have failed alkylating agents or fludarabine-based therapy. Additionally, it is approved in Canada for CLL patients who have not had any previous therapies. However, the benefit of alemtuzumab as frontline therapy is unclear with unsatisfactory impact on response in this setting [19]. In the updated results of the phase II CLL2O study, induction treatment with alemtuzumab combined with high dose dexamethasone demonstrated significantly improved ORR of 98% & 79% in untreated 17p- and relapsed 17p- patients respectively. High response rate was also seen in patients with fludarabine- refractory disease [20]. The safety and efficacy of alemtuzumab in combination with rituximab and PGG beta glucan was evaluated in a single arm trial [21]. Ten out of the 13 patients enrolled in the trial had high-risk parameters such as 17p-, 11q22-, unmutated IGHV and overexpression of ZAP70 and/or CD38. After 4 weeks of treatment, the ORR was 100% (7 CR, 1 CCR, 1 nPR, and 2 PR) in eleven evaluable patients. Grade 4 toxicity, mainly febrile neutropenia was observed in one patient. Therefore, combination of alemtuzumab with rituximab and PGG beta glucan seems well tolerated with acceptable toxicity profile (Table 1).

**Table 1:** Alemtuzumab in clinical trials

**Study agents**	**Other agents**	**Disease**	**Dosage**	**Clinical trials**	**No Pts**	**Response**	**Reference**
Alemtuzumab		High risk	30 mg	Phase II	93	CR:2%	[19]
						ORR:33%	
Alemtuzumab	Dexamethasone	Untreated	30 mg	Phase II	131	CR:19/4/3%	[20]
		Relapsed				ORR:98/79/70%	
		Refractory					
Alemtuzumab	Rituximab	High risk	30 mg	Phase I	13	CR:64%	[21]
	PGG beta glucan					ORR:100%	

Abbreviations. *ORR* overall response rate, *CR* complete response rate.

## New agents

### Bendamustine

Bendamustine is a unique alkylating agent with multifaceted mechanisms of action that is distinct from standard alkylating agents. This drug alone or in combination with other agents has been shown to have clinical efficacy in low grade non-Hodgkin’s lymphoma (NHL) including CLL. The results of the randomized phase III trial of 301 treatment-naive patients with CLL demonstrated a significantly higher ORR in patients treated with bendamustine compared with chlorambucil (59% versus 26%, P<0.0001). The median PFS was also found to be longer in the bendamustine arm (17.6 versus 5.7 months; P<0.0001) [22]. In addition, analysis of retrospective studies have also shown that primary therapy with bendamustine and rituximab is very active and well tolerated in heavily-pretreated and treatment-naïve newly diagnosed patients with CLL [23,24].

More importantly, Tadeschi et al. reported the results of a multicenter dose escalation study designed to evaluate the toxicity and response of bendamustine in combination with alemtuzumab in refractory/relapsed patients with CLL [25]. After a median of 4 cycles of treatment, the MTD of bendamustine was 70 mg/m^2^. Of 43 patients evaluated for response, the ORR was 70% (including 26% CR, 44% PRs), and 18% maintained a stable disease. Wierda and colleagues also demonstrated similar findings in another phase I/II dose escalation trial [26] (Table 2).

**Table 2:** Bendamustine in clinical trials

**Study agents**	**Other agents**	**Disease**	**Dose**	**Trials**	**No. of Pts**	**Response**	**Reference**
Bendamustine	chlorambucil	Untreated	100 mg/m^2^	Phase III	305	CR:29%	[22]
						ORR:68%	
Bendamustine	Rituximab	Untreated	60-100 mg/m^2^	Retrospective	142	CR:19%	[23]
		High risk				ORR:80%	
		Relapsed					
		Refractory					
Bendamustine	Rituximab	Untreated	133.6-165.9	Retrospective	217	CR>19%	[24]
Prednisone			mg/m^2^			ORR>83%	
Bendamustine	Alemtuzumab	Relapsed	50-70 mg/m^2^	Retrospective	50	CR:26%	[25]
		Refractory				ORR:70%	
Bendamustine	Fludarabine	High risk	20-50 mg/m^2^	Phase I/II	35	CR/CRi:26%	[26]
	Rituximab	Relapsed				ORR:71%	
		Refractory					

Abbreviations: *CR* complete remission, *CRi* CR with incomplete recovery of cytopenia; *ORR* overall response rate.

### Ofatumumab

Ofatumumab is a type 1 fully humanized G1 kappa anti-CD20 monoclonal antibody that binds to a different epitope on CD20 molecule compared with rituximab, and demonstrated higher complement-dependent cytotoxicity (CDC) and antibody-dependent cellular cytotoxicity (ADCC) in preclinical studies [27] (CAng, 2012). The antibody is licensed by the FDA for the treatment of patients with CLL refractory to fludarabine and alemtuzumab, at a total of 12 doses over a 28-week schedule. The benefit, dose and schedule of ofatumumab however, in frontline setting remains to be determined. In a recent open label trial, 77 previously untreated CLL patients were assigned to high-dose (2000 mg) or low-dose (1000 mg) once weekly treatment for eight weeks as induction therapy [28]. The ORR after primary treatment followed by maintenance at same dose every 2 months for 2 years was 55% in patients receiving high-dose therapy versus 36% in the low-dose therapy group.

Recent data indicated that the combination of rituximab and lenalidomide is very active in patients with refractory/relapsed CLL [29,30]. Lenalidomide augments and sensitizes CLL cells to rituximab-induced cell death. Therefore, the benefit of ofatumumab in combination with lenalidomide in CLL patients was subsequently investigated and recently addressed in two single arm trials. In the first trial, patients (n=36) received ofatumumab weekly for 4 weeks initially, then monthly during months 2–6 and every other month during months 7–24, and lenalidomide 10 mg on day 9 for 24 months [31]. Of thirty-four patients evaluated for response, an ORR of 68% was observed with a median duration of response of 22 months. The second phase I/II trial evaluated intracycle sequential treatment of ofatumumab plus lenalidomide in advanced, high risk CLL patients, and found the dosing and schedule to be well-tolerated [32]. The most common grade 3 and 4 toxicity was neutropenia, and was associated with infection in only one episode (Table 3).

**Table 3:** Ofatumumab in clinical trials

**Study agents**	**Other agents**	**Disease**	**Dosage**	**Clinical trials**	**No. of Pts**	**Response**	**Reference**
Ofatumumab		Untreated	2000/1000 mg	Phase II	77	CR:5/4%	[28]
						ORR:55/36%	
Ofatumumab	Lenalidomide	Relapsed	1000 mg	Phase II	36	CR:24%	[31]
						ORR:68%	
Ofatumumab	Lenalidomide	Relapsed	2000 mg	Phase II	17	ORR:43%	[32]
		Refractory				SD:21%	

Abbreviations: *ORR* overall response rate, *CR* complete remission, *SD* stable disease.

### Lenalidomide

Lenalidomide is a potent immunomodulatory drug with promising activity as salvage therapy for patients with relapsed/refractory CLL [33]. However, the safest and most effective dosing of lenalidomide in CLL patients remains to be determined. The agent is associated with occurrence of tumor flare reactions (TF), increased incidence of severe tumor lysis syndrome (TLS), and prolonged myelosuppression. Updated interim analysis results CLL-009 study demonstrated that all three starting doses (5 mg, 10 mg, or 15 mg on days 1–28 of each 28-day cycle) of lenalidomide were well tolerated in patients with refractory/relapsed CLL. The proportion of patients who developed ≥ grade 3 neutropenia and tumor flare reactions was 62% and 13% respectively [34].

Adding dexamethasone to lenalidomide with a view to mitigating its toxicity and enhance its antitumor activity was investigated in a recent open label trial [35]. Patients with previously untreated symptomatic disease received lenalidomide (5-25 mg) and dexamethasone 12 mg on days 1–7, 14, 21 of each 28 day cycle, both to a maximum of 18 cycles. The overall response rate was 59 percent (6 percent complete), and was reached after a median of 4 months. The rate of TF was considerably lower, and no TLS has been observed.

The safety and efficacy of lower dosing of lenalidomide has also been evaluated in combination with fixed doses of fludarabine and rituximab in 64 patients with advanced/symptomatic disease. The combination was found to be an acceptable alternative to FCR as the initial treatment for previously untreated patients [36] (Table 4).

**Table 4:** Lenalidomide in clinical trials

**Study agents**	**Other agents**	**Disease**	**Dosage**	**Clinical trials**	**No.of Pts**	**Response**	**Reference**
Lenalidomide		Relapsed	5/10/15 mg	Phase II	95	CR:2%	[34]
		Refractory				ORR:79%	
Lenalidomide	Dexamethasone	Untreated	5 mg	Phase II	18	CR:6%	[35]
						ORR:59%	
Lenalidomide	Fludarabine	Untreated	2.5 mg	Phase I/II	64	CR:11%	[36]
	Rituximab	Minimally				ORR:63%	
		treated					

Abbreviations: *ORR* overall response rate, *CR* complete remission.

### Ibrutinib

Ibrutinib is a potent irreversible inhibitor of Bruton’s tyrosine kinase (BTK), a critical enzyme in the B-cell receptor (BCR) signaling pathway that is essential for B-cell proliferation, survival, migration, and tissue homing [37]. Initial reports on the use of ibrutinib as a single agent found that it was well-tolerated and particularly active in patients with refractory/relapsed CLL patients, thus proving the rationale for further testing in ongoing trials [38].

The benefit-risk profile of ibrutinib as frontline therapy in treatment-naïve patients has also been determined. In a recent update, the ORR (among thirty-one previously untreated patients aged ≥65 years) was 71%, and the most common adverse effects were diarrhea, fatigue and rash [39]. Results of this trial suggest that ibrutinib could be a reasonable choice of treatment for older, treatment- naïve patients with CLL.

Ibrutinib exhibits antithrombotic properties, and is associated with ecchymosis/contusion, and rarely with serious bleeding in patients concurrently taking oral anticoagulation [40]. BTK is also a key component in the signaling of glycoprotein receptors, and ibrutinib may potentiate bleeding diathesis by interfering with this pathway and disrupting platelet adhesion and aggregation [41]. However, preliminary results from an ongoing open label phase II trial showed that ibrutinib did not significantly affect platelet function [42]. Von-willebrand factor (vWF) antigen levels, vWF activity, and FVIII levels which were mildly elevated prior to treatment decreased to normal on ibrutinib.

About 50% of high-risk CLL patients fail to achieve a meaningful response with ibrutinib and durable remissions are lacking [39]. A recent study however showed that high risk patients treated with ibrutinib plus rituximab had a higher ORR of 85%, and suggested additional development of ibrutinib for high-risk CLL patients [43] (Table 5).

**Table 5:** Ibrutinib in clinical trials

**Study agents**	**Other agents**	**Disease**	**Dosage**	**Clinical trials**	**No Pts**	**Response**	**Reference**
Ibrutinib		Relapsed	420 mg	Phase I	14	CR:14%	[38]
		Refractory				OR:79%	
		Untreated	420/840 mg	Phase Ib/II	116	CR:10/3/0%	[39]
						ORR:71/67/50%	
Ibrutinib		Relapsed/Refractory					
		High risk					
Ibrutinib	Rituximab	High risk	420 mg	Phase II	40	ORR:85%	[43]

Abbreviations: *ORR* overall response rate, *OR* overall response, *CR* complete remission.

### Idelalisib (GS-1101, CAL-101)

Idelalisib, also known as GS-1101 or CAL-101, is a first-in-class specific inhibitor of the phosphoinositide-3 kinase (PI3K) delta isoform with potent apoptotic activity against leukemic CLL cells.

Furman et al. have reported that idelalisib as a single salvage therapy offered modest response rates in heavily pretreated patients with relapsed/refractory CLL [44,45]. However, in a more recent report, idelalisib (I) in combination with rituximab (R) and/or bendamustine (B) in relapsed/refractory CLL was associated with improved ORR of 78, 82, and 87 percents for IR, IB, and IRB regimens respectively [46]. Additional clinical trials are ongoing to establish these regimens as optimal second-line treatments in patients with CLL (Table 6).

**Table 6:** Idelalisib in clinical trials

**Study agents**	**Other agents**	**Disease**	**Dosage**	**Clinical trials**	**No. Pts.**	**Response**	**Reference**
Idelalisib		Relapsed	150 mg	Phase I	54	ORR:26%	[44,45]
		Refractory					
Idelalisib	Rituximab	Relapsed	150 mg	Phase I	51	ORR:74/82/87%	[46]
	Bendamustine	Refractory				1 year PFS:74/88/87%	

Abbreviations: *ORR* overall response rate, *PFS* progression-free survival.

### Veltuzumab

Veltuzumab is another second-generation anti-CD20 monoclonal antibody that binds selectively and irreversibly to CD20 molecule [9]. It is fully humanized and attacks leukemic B-cell via with significant CDC and ADCC activities. Preclinical studies with veltuzumab demonstrated similar antigen-binding site as rituximab but with enhanced binding avidity [9]. In a recent report, prolonged subcutaneous administration of veltuzumab demonstrated excellent anti-CLL activity in patients with previously untreated and relapsed/refractory CLL with an acceptable tolerability profile (no grade 3–4 events) [47]. The levels of circulating leukemic cells was observed to be significantly reduced, suggesting further studies of this agent in combination with chemotherapy to optimize response.

### XmAb5574

CD19 monclonal antibodies are being explored for clinical applications [48]. XmAb5574 is a novel humanized IgG1 anti-CD19 monoclonal antibody with a modified constant fragment (Fc)–domain designed to enhance its binding avidity. In preclinical studies, XmAb5574 has shown antitumor activity including direct cytotoxicity, ADCC and antibody-dependent cellular phagocytosis against leukemic CLL cells [49]. Unlike other anti-CLL monoclonal antibodies, XmAb5574 demonstrated no CDC activity. In the first clinical testing, Woyach and colleagues reported that XmAb5574 showed tolerable toxicity profile and preliminary evidence of antitumor activity in high-risk patients with relapsed/refractory CLL [50]. Based on the results of this study, a phase 2 study of this agent in patients with CLL and other B- cell malignancies is encouraged.

### Navitoclax (ABT-263)

Navitoclax (also known as ABT-263) is a potent BH3-mimetic inhibitor of Bcl-2 family of pro-survival proteins including Bcl-xL, Bcl-2 and Bcl-w [51,52]. Roberts and colleagues have demonstrated the efficacy and feasibility of second-line navitoclax in patients with relapsed/refractory disease [53]. Efficacy of navitoclax in association with rituximab has also been evaluated in a recent study of 118 previously untreated patients which included those with high-risk features [54]. At a median follow-up of 12 weeks, the response (CR and PR) rates were 35% for induction rituximab alone, 55% for induction rituximab plus navitoclax, and 70% for induction rituximab plus navitoclax followed by maintenance navitoclax. This combination although active is associated with substantial hepatotoxicity.

### Dasatinib

Dasatinib is a novel, oral multikinase inhibitor that blocks the activity of several protein kinases, including Abl, Src, c-Kit, and ephrin receptors. It is approved by the FDA for first line use in patients with CML and Ph+ ALL, and for similar patients who are intolerant or refractory to imatinib [55,56]. Data from preclinical studies showed dasatinib sensitizes leukemic CLL cells to fludarabine-induced cell cycle arrest and apoptosis by inhibiting Src signaling [57,58].

Clinical efficacy and tolerability of dasatinib in combination with fludarabine in patients with refractory CLL was presented at the 2012 annual ASH meeting [59]. Combined dasatinib plus fludarabine treatment induced durable responses with an acceptable toxicity profile in heavily-pretreated CLL patients. Additional studies are required to establish this combination as a salvage therapy option in patients with relapsed/refractory CLL.

### TRU-016

TRU-016 is a novel humanized small modular immunopharmaceutical (SMIP) protein designed to target CD37, a molecule that is highly expressed on normal and leukemic B-cells [60]. It possesses potent direct cytotoxicity and antibody-dependent cellular cytotoxicity against CLL cells that are superior to rituximab. TRU-016 and bendamustine has been used in the management of relapsed CLL. Promising results showed the regimen was effective in heavily-pretreated patients with manageable adverse effects [61]. The reported ORR was 100% including 4 patients obtaining a CR. Another phase 1b/2 study of TRU-016/bendamustine versus bendamustine alone is currently ongoing (Clinical Trial ID: http://clinicaltrials.gov/NCT01188681).

### Bortezomib

Bortezomib is a dipeptide, boronic acid inhibitor of the proteasome approved by the FDA as first line treatment option for patients with multiple myeloma. As a single agent, bortezomib has also displayed promising antitumor effects in CLL lymphocytes [62]. More so, *in- vitro* studies of bortezomib plus romidepsin (histone deacetylase inhibitor) at low concentrations demonstrated enhanced antiproliferative and proapoptotic activity in leukemic CLL cells [63].

The synergistic effects of bortezomib and romidepsin were confirmed in an ongoing phase 1 trial in patients with relapsed/refractory CLL, showing a disease control rate of up to 55%. Myelosuppression and soft tissue infections were the most serious adverse events [64].

### Alvespimycin (17-DMAG, KOS-1022)

Alvespimycin, a synthetic derivative of the antibiotic geldanamycin, is a potent inhibitor of heat shock protein 90 (Hsp90). It regulates genes and proteins involved in the proliferation and survival of CLL cells. In a phase I dose-escalation study, intravenous alvespimycin produced no DLTs and the MTD was determined to be 24 mg/m^2^[65]. Of fourteen patients evaluated for response, 3 demonstrated stable disease after 2 cycles of treatment.

## Conclusions and future directions

Bendamustine and rituximab is being compared with FCR as a front-line therapy for CLL. Aggressive regimens are being explored for relapsed/refractory or high risk CLL. Nevertheless, CLL remains an incurable disease at this time. In particuar, the long-term survival of CLL patients with poor prognosis is still unsatisfactory. Novel agents discussed in this article appear encouraging. Additional targets are being explored [66-68]. By integrating these novel agents with conventional approaches, significant progress may become reality in the near future.

## Competing interests

The authors declare that they have no competing interests.

## Authors’ contributions

XPZ developed the concept and report design. All authors equally participated in the drafting and revisions of this article, and have read and approved the final manuscript.
